# Extracellular vesicles with ubiquitinated adenosine A_2A_ receptor in plasma of patients with coronary artery disease

**DOI:** 10.1111/jcmm.14564

**Published:** 2019-08-24

**Authors:** Jean Ruf, Donato Vairo, Franck Paganelli, Régis Guieu

**Affiliations:** ^1^ Center for CardioVascular and Nutrition Research (C2VN) INSERM, INRA and Aix‐Marseille University Marseille France; ^2^ Department of Cardiology North Hospital Marseille France; ^3^ Laboratory of Biochemistry Timone University Hospital Marseille France

**Keywords:** adenosine A_2A_ receptor, coronary artery disease, extracellular vesicles, homocysteine, ubiquitin

## Abstract

Extracellular vesicles (EV) can transfer cellular molecules for specific intercellular communication with potential relevance in pathological conditions. We searched for the presence in plasma from coronary artery disease (CAD) patients of EV containing the adenosine A_2A_ receptor (A_2A_R), a signalling receptor associated with myocardial ischaemia and whose expression is related to homocysteine (HCy) metabolism. Using protein organic solvent precipitation for plasma EV preparation and Western blotting for protein identification, we found that plasma from CAD patients contained various amounts of EV with ubiquitin bound to A_2A_R. Interestingly, the presence of ubiquitinated A_2A_R in EV from patients was dependent on hyperhomocysteinemia, the amount being inversely proportional to A_2A_R expression in peripheral mononuclear cells in patients with the highest levels of HCy. CEM, a human T cell line, was also found to released EV containing various amounts of ubiquitinated A_2A_R in stimulated conditions depending on the hypoxic status and HCy level of culture medium. Together, these data show that ubiquitinated A_2A_R‐containing EV circulate in the plasma of CAD patients and that this presence is related to hyperhomocysteinemia. A_2A_R in plasma EV could be a useful tool for diagnosis and a promising drug for the treatment of CAD.

## 1 INTRODUCTION

Extracellular vesicles (EV) such as exosomes and microvesicles are bi‐lipid membranous vesicles with endocytic origin that are released by many cell types including immune, endothelial and mesenchymal stem cells, erythrocytes and platelets.^1^ EV participate in intercellular communication by carrying and delivering cargo including proteins, lipids, miRNA and mRNA specific to the type of cell from which they originate.^2^ EV are key mediators of a process now thought to be a form of intercellular signalling that impacts the physiology of cells, tissues and organs.^3^ EV are released constitutively or after stimulation and taken up by other cells via membrane fusion or ligand‐receptor interactions.^4^ Due to their ability to trap their cargo and circulate freely in body fluids, EV are natural sources of non‐invasive diagnostic and prognostic biomarkers that may also be used as vehicles of targeted therapy for tumour progression, neurodegeneration, autoimmune disorders and other human diseases.^5^ In cardiovascular disease, EV represent one of the most intensely studied and rapidly growing areas of research.^6^, ^7^ EV were shown to exert diverse and sometimes discordant biological effects in different studies related to cardiovascular disease. For example, EV can play an atheroprotective or atherogenic role in several conditions accompanying atherosclerosis.^8^

Adenosine greatly impacts the cardiovascular system via four specific G protein‐coupled receptors, named respectively A_1_, A_2A_, A_2B_ and A_3_. Among them, the A_2A_ receptor (A_2A_R) is strongly expressed in coronary cells and its activation increases coronary blood flow,^9^ partly through the production of cAMP in target cells.^10^ A_2A_R from patients with coronary artery disease (CAD) is poorly expressed and, consequently, produces low level of cAMP, two characteristics that are associated with myocardial ischaemia, as documented by positive exercise stress testing or reduced flow reserve.^11^, ^12^, ^13^ The down‐regulation of A_2A_R expression in CAD patients is related to the homocysteine (HCy) metabolism via its degradation product H_2_S.^14^ A_2A_R expressed on peripheral blood mononuclear cells (PBMC) of CAD patients reflect coronary tissue expression showing the systemic nature of the adenosinergic signalling.^15^

Circulating EV can be considered as a reserve of functional G protein‐coupled receptors as previously suggested from data obtained on a mouse model of heart cellular stress for angiotensin II type 1 receptor.^16^ Taking into account the major role of A_2A_R in cardiovascular disease and the potential contribution of circulating EV in delivering cell receptor from donor to target cells, we searched for the presence of A_2A_R in EV from plasma of patients with CAD and culture supernatant of human lymphoblastoid T cells cultured in CAD‐like conditions.

## 2 MATERIALS AND METHODS

### 2.1 Human materials

Fourteen patients (11 men and three women, 56‐58 years old) with angiographically documented CAD were included in this pilot study (Table 1). The first group consisted of eight patients selected blind and the second group was six patients with moderate hyperhomocysteinemia. Controls were eight healthy individuals (six men and two women, 56‐64 years old) with a normal level of HCy (Table 1) recruited from the research laboratory or hospital staff, without medical treatment or history of cardiovascular disease. The study was conducted in compliance with the principles of the Declaration of Helsinki and approved by the Ethics Committee for Human Research of our University Hospital. All participants provided written informed consent to participate.

**Table 1:** HCy levels of healthy individuals and CAD patients

Healthy individuals				Unselected CAD patients				CAD patients with moderate hyperhomocysteinemia			
Ref	Age	Sex	HCy	Ref	Age	Sex	HCy	Ref	Age	Sex	HCy
A	59	M	10.7 ± 0.2	1	56	F	13.5 ± 0.5	9	57	M	31.4 ± 1.6
B	60	M	9.3 ± 0.1	2	60	M	14.8 ± 1.4	10	59	M	24.1 ± 0.9
C	56	M	9.2 ± 0.3	3	56	M	13.1 ± 0.8	11	63	M	38.7 ± 2.1
D	57	F	8.7 ± 0.5	4	61	M	32.1 ± 2.3	12	57	M	27.7 ± 1.3
E	64	M	8.9 ± 0.7	5	57	M	16.7 ± 0.7	13	62	M	32.5 ± 1.5
F	59	F	10.9 ± 0.7	6	68	F	24.1 ± 1.2	14	65	F	23.8 ± 1.9
G	63	M	9.1 ± 0.6	7	57	M	11.1 ± 0.9				
H	56	M	10.4 ± 1.0	8	63	M	18.6 ± 1.2				

Note

Age (Y), Sex (Male/Female), HCy (µmol/L); Mean ± SD. Normal level of HCy: <12 µmol/L; Mild Hyperhomocysteinemia: 12‐20 µmol/L; Moderate Hyperhomocysteinemia: 21‐100 µmol/L.

Abbreviation: Ref: Reference Letter for Healthy Subjects and Reference Number for CAD patients.

Plasma and PBMC were obtained from blood collected by venipuncture at the brachial vein in citrate tube and cell preparation tube (CTP) for the separation of mononuclear cells (Vacutainer, Becton‐Dickinson), respectively, according to the manufacturer's instructions.

Extracellular vesicles were isolated from fresh plasma by a simple, rapid and reliable solvent‐based protein precipitation method as previously described.^17^ Briefly, 200 µL of plasma was mixed with 800 µL cold acetone (−20°C) in 1.5 mL conical tube and centrifuged at 3000 × g for 1 minute. After centrifugation, 500 µL of EV‐containing supernatant was freeze at −20°C and dried in a Savant SpeedVac concentrator (Thermo Fisher Scientific).

Extracellular vesicles and PBMC were analysed by Western blotting as previously reported.^11^, ^12^, ^13^, ^14^, ^15^ Briefly, freeze‐dried EV corresponding to 100 µL of plasma and cell pellets corresponding to 0.25 × 10^6^ PBMC were solubilized in 15 µL Laemmli sample buffer (BioRad) with 5% SDS, 5% 2‐mercaptoethanol and a complete set of protease inhibitors (Roche), heated for 5 minutes at 95°C and loaded on 12% SDS‐PAGE minigel (BioRad). Prestained molecular weight markers (BioRad) have always been loaded onto the gel. After electrophoresis, separated proteins were blotted onto nitrocellulose membrane. Protein detection was conducted using an appropriate primary antibody: anti‐CD63 and anti‐CD9 (EXOAB‐KIT, Ozyme), anti‐ubiquitin (clone 6C1, Sigma‐Aldrich) and anti‐A_2A_R (Adonis,^18^ CliniSciences). Blots were then revealed using phosphatase alkaline‐labelled secondary antibody by a BCIP^®^/NBT‐Purple liquid substrate system for membranes, (B3679, Sigma‐Aldrich). Densitometric quantification of the blot was performed using the ImageJ software (https://imagej. nih.gov). Briefly, a scanned blot image (in grey scale and TIFF format) was imported into ImageJ, areas of interest were selected and plots of pick profiles were generated. Lines were drawn to select the peaks of interest, and the peak areas were integrated and converted into pixel intensities. Results were given as per cent of total pixels of all the relevant bands on a same migration line on the blot. The protein load was controlled by the reproducibility of the results, Western blots being done in triplicate.

### 2.2 Homocysteine assay

Total HCy from the human plasma was quantified with the liquid chromatography‐tandem mass spectrometry Clinmass^®^ apparatus using the dedicated kit (Homocysteine in plasma/serum, Recipe) according to the manufacturer's instructions. Results (in µmol/L) are the mean ± SD of duplicates.

### 2.3 CEM T cell line

CEM, a human lymphoblastoid T cell line^19^ was cultured in RPMI 1640 medium supplemented with 2 mmol/L l‐glutamine, 10% foetal calf serum (cleaned for cell debris and aggregates by ultracentrifugation and filtration through a 0.22 µm sterile membrane) and 100 U/mL penicillin + 100 µg/mL streptomycin at 37°C under 5% CO_2_. Cells were seeded in 75‐cm^2^ flasks (0.5 × 10^6^ cells/mL, 50 mL/flask) and stimulated using phorbol myristate acetate (PMA, 50 ng/mL) and phytohemagglutinin (PHA, 5 µg/mL) for 24 hours in control condition. As previously reported in this cellular model, hypoxia condition was achieved by adding 100 µmol/L CoCl_2_ to the culture medium and the effect of HCy on cells in hypoxia was obtained by adding 200 µmol/L HCy just prior to the addition of CoCl_2_.^14^

After 24 hours incubation, cells cultured in the three conditions (Control, Hypoxia, Hypoxia + HCy) were separated from supernatants by centrifugation at 3000 × g for 15 minutes. Cells were rinsed in phosphate‐buffered saline, pH 7.3, counted and aliquoted for A_2A_R expression assay. EV were prepared from the culture supernatant by a dedicated method using the ExoQuick‐TC reagent (System Biosciences) according to the manufacturer's instructions. Briefly, culture supernatant (10 mL) and 2 mL reagent were mixed well by inverting into a 15 mL tube. After overnight incubation at 4°C, the mixture was centrifuged at 1500 × g for 30 minutes and the supernatant was discarded. Residual supernatant was removed by additional centrifugation and careful aspiration to not disturb the pelleted material. EV was solubilized in 100 µL Laemmli sample buffer (BioRad) with 5% SDS, 5% 2‐mercaptoethanol and a complete set of protease inhibitors (Roche) and heated for 5 minutes at 95°C. EV from culture supernatant and CEM T cells were analysed by Western blotting as described above for the human material.

Cell viability was monitored using the 3‐(4,5‐dimethyl‐2‐thiazolyl)‐2,5‐diphenyl‐2H‐tetrazolium bromide (MTT) assay as previously described.^14^ MTT (0.5 mg in 100 µL of PBS, pH 7.3) was added to 24‐well plates containing 0.5 × 10^6^ cells/mL cultured in the three conditions (Control, Hypoxia, Hypoxia + HCy) 3 hours prior to the end of the 24 hours incubation period. After treatment, cells were pelleted (10 000 × g for 5 minutes) and supernatants were discarded. The insoluble violet formazan crystals associated with the cell pellets were dissolved into pure dimethyl sulfoxide and absorbance was measured at 550 nm. Results are the mean ± SD of duplicates.

## 3 RESULTS

### 3.1 Presence of ubiquitinated A_2A_R in EV of CAD patients

**Figure 1:**
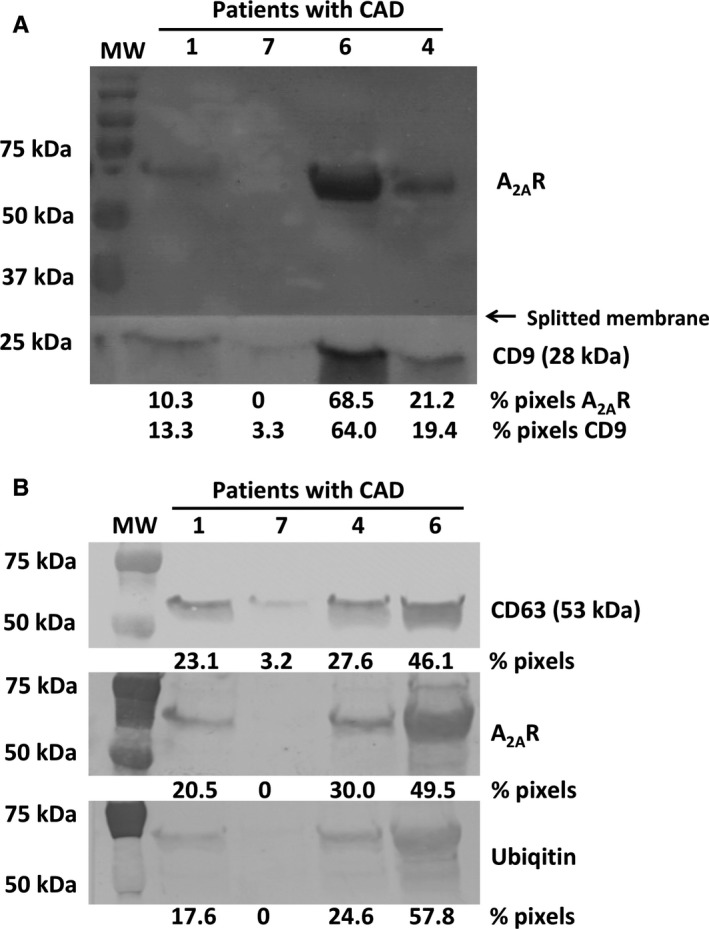
Presence of ubiquitinated A_2A_R in EV from plasma of CAD patients. Western blot of EV from plasma of four CAD patients was splitted in two parts and separately revealed using anti‐A_2A_R (upper part) and anti‐CD9 (lower part) primary antibody (A). Western blots of EV from four CAD patients were revealed using (from top to bottom) anti‐CD63, anti‐A_2A_R and anti‐ubiquitin primary antibody (B). Molecular weight markers are on the left. To compare, blots obtained with each primary antibody are mounted on the figure and are representative of triplicates

First, we tested plasma from four patients with CAD (Reference Numbers 1, 4, 6 and 7) for the presence of EV carrying A_2A_R. EV were isolated from plasma using acetone to remove proteins by precipitation leaving purified EV suspended in the liquid phase.^17^ Freeze‐dried EV were submitted to Western blot procedure using Adonis, a monoclonal antibody to the human A_2A_R 18 widely used in previous studies 11, 12, 13, 14, 15 and anti‐tetraspanin CD9 as a biomarker of EV with a low molecular weight (28 kD) migrating at high distance from the A_2A_R to test both in same lanes of gel. Intriguingly, we found using Adonis various amounts of a band (intensity from 0% to 68.5% pixels depending of the patient) that migrated to a higher position than expected (45 kD for cellular A_2A_R) between the 75 and 50 kD markers in three to four patients (Figure 1A). We found CD9 bands displaying similar intensities (from 3.3% to 64.0% pixels) to those obtained for the A_2A_R. The negative patient for A_2A_R probably contained a low level of receptors, which corresponded to a low level of EV in plasma as judged by the faint intensity of the CD9 band (3.3% pixels) and consequently had undetectable A_2A_R in our experimental conditions (Figure 1A).

Next, we performed another set of Western blots using preparations of EV from the same four plasma samples. The use of a different anti‐tetraspanin marker CD63 (mol wt of 53 kD) gave a band intensity gradation similar to that obtained with CD9 and confirmed that the plasma level of A_2A_R in CAD patients was related to their EV content ranking patients from lowest to highest as follows: Reference Numbers 7,1, 4 and 6 (Figure 1B). Looking for a plausible post‐translational process that could have increased the molecular weight of the A_2A_R in the context of EV trafficking we used an anti‐ubiquitin antibody, which revealed the same heavy A_2A_R bands as Adonis with a similar intensity gradation between patients, as previously described (Figure 1B). Blotted bands of ubiquitinated A_2A_R migrated above the CD63 bands. Together, these data suggested that exosomal A_2A_R contained at least two molecules of ubiquitin (mol wt 8 kD) raising the molecular weight of A_2A_R from 45 to 61 kD.

### 3.2 Comparison of EV and cell expression of A_2A_R

**Figure 2:**
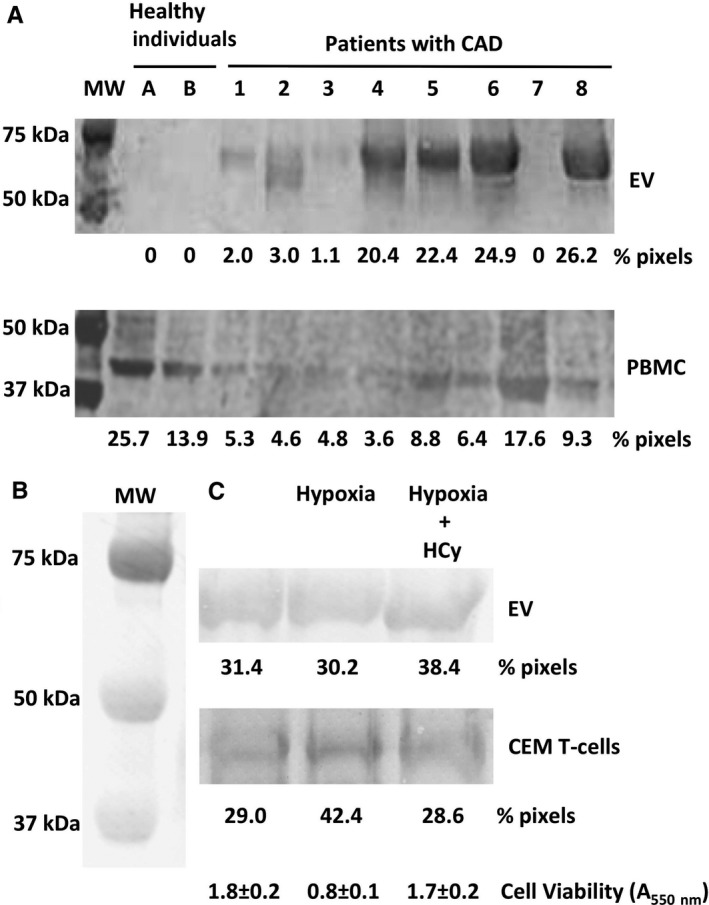
Comparison of EV and cell expression of A_2A_R. Western blots of EV (upper) and PBMC (lower) from eight CAD patients and two healthy individuals (A). Western blots of EV (upper) and CEM T cells (lower) from cells cultured in three conditions: Control, Hypoxia, Hypoxia + HCy (B). Blots were revealed using anti‐A_2A_R primary antibody. Molecular weight markers are on the left. To compare, blots are mounted on the figure and are representative of triplicates. Blotted bands were quantified, and results are given in % of total pixels. At the bottom of the figure, CEM cell viability after 24 h culture in the three conditions is given in absorbance at 550 nm (mean ± SD)

Second, we examined A_2A_R content both in EV and PBMC from eight unselected CAD patients (Reference Numbers 1‐8) and two healthy individuals(Reference Letters A and B). To compare the A_2A_R level in EV from 100 µL plasma to that contained in 0.25 × 10^6^ PBMC, Western blots were made in triplicates, each giving a similar result. Densitometric quantification of representative blots was performed and expressed as % of total pixels (Figure 2A). Intensity of the patient bands varied from 0% to 26.2% pixels in EV and from 3.6% to 17.6% pixels in PBMC. EV bands from patients 4 and 6 were among the strongest (20.4 and 24.9% pixels, respectively) when their cell counterparts from PBMC were among the weakest (3.6 and 6.4% pixels, respectively). Patients 7 as the two healthy individuals A and B gave inverted results, that is no band for A_2A_R from EV as compared to strongest bands for A_2A_R from PBMC (17.6, 25.7 and 13.9% pixels, respectively). Relative levels in the EV and PBMC compartments were almost similar for other patients, that is, low in patients 1, 2 and 3 and high in patients 5 and 8. Interestingly, patients 4 and 6 had the highest levels of HCy (32.1 ± 2.3 and 24.1 ± 1.2 µmol/L, respectively) indicating moderate hyperhomocysteinemia. The other patients had mild hyperhomocysteinemia whereas patients 7 and healthy individuals had normal level of HCy (Table 1).

We then confirmed the presence of heavy A_2A_R (61 kD) in EV released by stimulated CEM T cells in culture medium and prepared using a commercial kit (Figure 2B). Western blots were performed as above using EV from 10 mL of culture supernatant and 0.25 × 10^6^ CEM T cells. Maximal release of A_2A_R in EV (38.4% pixels) was observed in cells cultured in hypoxia + HCy conditions. A_2A_R expression in CEM T cells increased under stress hypoxia (42.4% pixels) and returned to control value in the presence of HCy (28.6% pixels). Hypoxia stress was confirmed by about fifty per cent of cell loss as compared to control (A_550 nm_ = 0.8 ± 0.1 vs 1.8 ± 0.2), which was reversed by HCy (A_550 nm_ = 1.7 ± 0.2) as assessed by the MTT assay.^14^ The highest content of A_2A_R in EV (38.4% pixels) matched with the lowest cellular content of A_2A_R (28.6% pixels) and, conversely, the lower one in EV was the highest in cells (30.2% vs 42.4% pixels, respectively).

### 3.3 Relationship between A_2A_R export in EV and hyperhomocysteinemia

**Figure 3:**
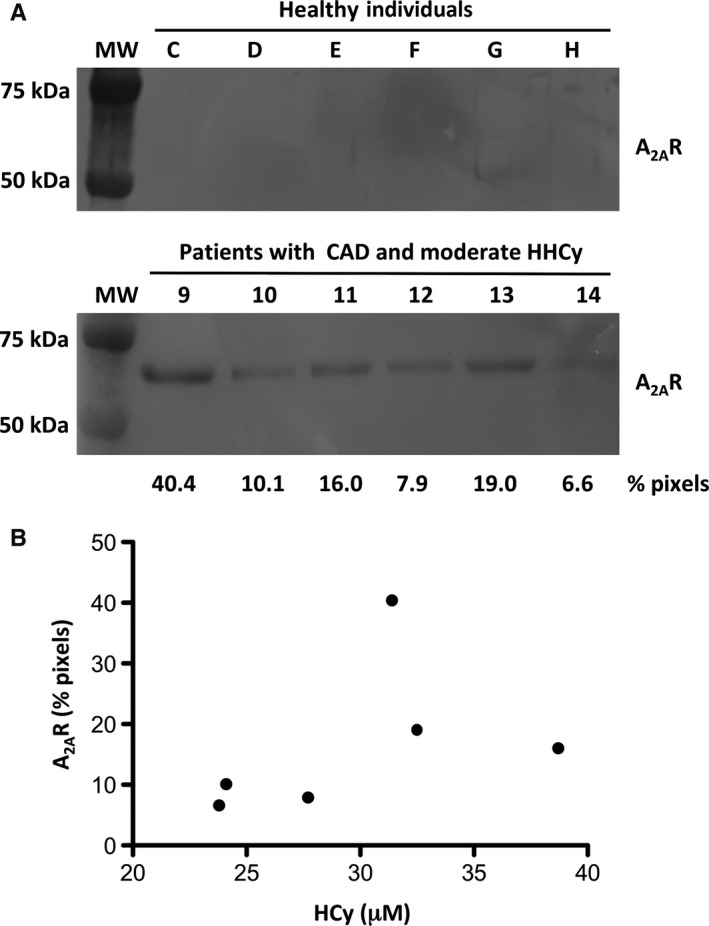
Relationship between A_2A_R export in EV and hyperhomocysteinemia (HHCy). Western blots of EV from six healthy individuals (upper) and six CAD patients with moderate hyperhomocysteinemia (lower). Blots were revealed using anti‐A_2A_R primary antibody. Molecular weight markers are on the left. Blots are representative of triplicates. Blotted bands were quantified and results are given in % of total pixels (A). Scatter plot to display the correspondence of A_2A_R levels in EV of CAD patients (% pixels) with their HCy levels (µmol/L) given in Table 1. Each point represents a patient (B)

Finally, to further explore the relationship between the A_2A_R export via EV and hyperhomocysteinemia, we compared on a same blot the A_2A_R content of the EV from 6 healthy individuals (Reference Letters C‐H) with normal level of HCy taken as controls with that of 6 CAD patients (Reference Numbers 9‐14) with moderate hyperhomocysteinemia (Table1). The results shown in Figure 3 confirmed the previous data. The control samples lacked A_2A_R, but those from CAD patients with moderate hyperhomocysteinemia all expressed the A_2A_R band of a pixels intensity of 6.6 to 40.4% (Figure 3A). The scatter plot in Figure 3B shows the relationship between A_2A_R levels in EV of CAD patients and their HCy levels greater than 20 µmol/L. The patients 10, 12 and 14 with the lowest HCy levels gave the A_2A_R bands with the lowest intensities and the patients 9, 11 and 13 with the highest HCy levels had the most A_2A_R in their EV.

## 4 DISCUSSION

We report here for the first time the presence of ubiquitinated A_2A_R in EV isolated from plasma of CAD patients. Ubiquitinated A_2A_R were also found in EV released in the culture supernatant of a stimulated human T cell line.

Plasma contains a mix of EV with different sizes derived from many different cells in varying proportions. Sorting of many membrane proteins into EV coincides with their association with tetraspanin membrane proteins.^20^ We found that A_2A_R expression in EV was related to a similar expression of EV assessed by CD9 and CD63 tetraspanins.

We also found here that A_2A_R in EV was ubiquitinated. Ubiquitin is a highly conserved 76 amino acid polypeptide that is covalently attached to substrate proteins via a lysine residue. Ubiquitination is a reversible modification and ubiquitin moieties can be removed from the substrate protein by a family of deubiquitinating enzymes.^21^ G protein‐coupled receptors that undergo agonist‐induced ubiquitination are usually internalized and targeted for degradation in lysosomes. However, after deubiquitination they can be redirected to the resensitization pathway and recycled back to the cell surface.^22^ The addition of a single ubiquitin to a substrate is defined as monoubiquitination and is implicated in various functions including endocytosis of plasma membrane proteins and sorting of proteins to the multivesicular body.^23^ Moreover, several lysine residues in the substrate can be tagged with single ubiquitin molecules, giving rise to multiple monoubiquitination.^24^ Here, the ubiquitinated A_2A_R migrated in SDS‐PAGE to approximately 61 kD suggesting that it contained at least two molecules of ubiquitin. Given the presence of two potential ubiquitination sites on lysine residues 315 and 391 in the intracellular part of A_2A_R according to an online calculator (Ubpred.org), A_2A_R could be monoubiquitinated at two sites.

EV with A_2A_R appeared strongly released in blood concomitantly to down‐regulation of cell surface A_2A_R expression in CAD patients with moderate hyperhomocysteinemia. This relationship was confirmed in cellulo. These results are in agreement with our previous observation that A_2A_R cell expression in hypoxia condition is down‐regulated by H_2_S produced from HCy via the transsulfuration pathway.^14^ In adenosinergic signalling, exoenzymes CD39 and CD73 are expressed in cancer exosomes where they produce adenosine, which inhibits T cell functions in tumour environment.^25^ In CAD, one could hypothesize that A_2A_R circulate from donor cells to target cells as a salvage pathway to be stimulated by adenosine when vasodilation is required for oxygen delivery in failing artery tissues. This mechanism may explain the low expression of A_2A_R with spare receptor characteristics (EC_50_ < K_D_) found in PBMC from CAD patients presenting a major cardiovascular risk 12, 13 by a release from the cells into the blood of EV carrying A_2A_R, constituting a circulating pool of receptors.

Here, moderate hyperhomocysteinemia in CAD patients was still associated with release of A_2A_R in EV, which questioned the molecular basis of this association. HCy can affect intracellular signalling by acting on the mitogen‐activated protein kinase pathways 26, 27 and cell signalling can be related to endocytosis and endosomal trafficking.^28^ We therefore assume that HCy could act on the ubiquitination process so that A_2A_R do not undergo proteosomal degradation but are exported into exosomes. In this case, hyperhomocysteinemia would favour the salvage pathway described above.

Exosomes contain ubiquitinated proteins that can serve as markers of exosomes 29 and ubiquitin plays a major role in the endosomal trafficking leading to exosomal release.^30^ Otherwise, ubiquitination controls cell surface expression of MHC class II molecules in dendritic cells 31 and the ubiquitin‐specific protease USP4 was reported to regulate the cell surface level of the A_2A_R in HEK293 transfected cells.^32^ Multiple monoubiquitins could confer resistance to the action of deubiquitinating enzymes that would otherwise result in rapid elimination of a single ubiquitin, thereby promoting receptor recycling.^33^ Given all these considerations, the putative presence here of bi‐monoubiquitinated A_2A_R in EV delivered in plasma from CAD patients with hyperhomocysteinemia and surpernatant from hypoxic CEM cells under HCy treatment suggests a pivotal role of the ubiquitination process in cell export of A_2A_R under pathological conditions.

In conclusion, our data show that plasma from CAD patients with hyperhomocysteinemia contains EV carrying ubiquitinated A_2A_R. Ubiquitin might function here as a tag for A_2A_R delivery into the blood. From a clinical point of view, EV with A_2A_R constitute a potential diagnostic tool in CAD and a promising treatment for ischemic tissue.

## CONFLICT OF INTEREST

None.

## AUTHORS' CONTRIBUTION

JR designed the study. FP provided the human samples. DV performed the experiments. JR, DV, FP and RG analysed the data. JR wrote the article with input from all authors.

## Data Availability

The data that support the findings of this study are available from the corresponding author upon reasonable request.
